# Identification of putative transcriptomic biomarkers in irritable bowel syndrome (IBS): Differential gene expression and regulation of *TPH1* and *SERT* by vitamin D

**DOI:** 10.1371/journal.pone.0275683

**Published:** 2022-10-20

**Authors:** Aleksandra Grozić, Keaton Coker, Christopher M. Dussik, Marya S. Sabir, Zhela Sabir, Arianna Bradley, Lin Zhang, Jin Park, Steven Yale, Ichiro Kaneko, Maryam Hockley, Lucinda A. Harris, Tisha N. Lunsford, Todd R. Sandrin, Peter W. Jurutka

**Affiliations:** 1 School of Mathematical and Natural Sciences, Arizona State University, Phoenix, AZ, United States of America; 2 Biodesign Institute, Arizona State University, Tempe, AZ, United States of America; 3 Department of Medicine, University of Central Florida College of Medicine, Orlando, FL, United States of America; 4 Department of Basic Medical Sciences, University of Arizona College of Medicine, Phoenix, AZ, United States of America; 5 Mayo Clinic Division of Gastroenterology & Hepatology, Alix School of Medicine, Mayo Clinic, Scottsdale, AZ, United States of America; 6 Julie Ann Wrigley Global Futures Laboratory, Arizona State University, Tempe, AZ, United States of America; Medical University of Gdańsk, POLAND

## Abstract

Irritable bowel syndrome (IBS) is one of the most common gastrointestinal disorders and affects approximately 4% of the global population. The diagnosis of IBS can be made based on symptoms using the validated Rome criteria and ruling out commonly occurring organic diseases. Although biomarkers exist for “IBS mimickers” such as celiac disease and inflammatory bowel disease (IBD), no such test exists for IBS. DNA microarrays of colonic tissue have been used to identify disease-associated variants in other gastrointestinal (GI) disorders. In this study, our objective was to identify biomarkers and unique gene expression patterns that may define the pathological state of IBS. Mucosal tissue samples were collected from the sigmoid colon of 29 participants (11 IBS and 18 healthy controls). DNA microarray analysis was used to assess gene expression profiling. Extraction and purification of RNA were then performed and used to synthesize cDNA. Reverse transcriptase quantitative polymerase chain reaction (RT-qPCR) was employed to identify differentially expressed genes in patients diagnosed with IBS compared to healthy, non-IBS patient-derived cDNA. Additional testing probed vitamin D-mediated regulation of select genes associated with serotonergic metabolism. DNA microarray analyses led to the identification of 858 differentially expressed genes that may characterize the IBS pathological state. After screening a series of genes using a combination of gene ontological analysis and RT-qPCR, this spectrum of potential IBS biomarkers was narrowed to 23 genes, some of which are regulated by vitamin D. Seven putative IBS biomarkers, including genes involved in serotonin metabolism, were identified. This work further supports the hypothesis that IBS pathophysiology is evident within the human transcriptome and that vitamin D modulates differential expression of genes in IBS patients. This suggests that IBS pathophysiology may also involve vitamin D deficiency and/or an irregularity in serotonin metabolism.

## Introduction

Irritable bowel syndrome (IBS) is a disorder of gut-brain interaction (DGBI) that affects approximately 4% of the global population but estimates vary based on nonuniform study methodology [1]. Patients with IBS often display symptoms such as altered stool forms, constipation, bloating, and abdominal pain with overlapping co-morbidities including depression, anxiety, chronic fatigue, insomnia, sexual dysfunction, and fibromyalgia, collectively impairing quality of life [2, 3]. A 2013 systematic review estimated the direct and indirect health care costs of IBS to be between $22–30 billion [4].

Symptom-based criteria along with the judicious use of testing have improved the diagnosis of IBS but the average patient has often seen several physicians and undergone extensive costly testing before clearly being told they had IBS. Additionally, the lack of reliable biomarkers to help make an accurate diagnosis has added to the challenge of arriving at a diagnosis in a timely fashion [5–7]. The diagnostic tool, Rome III criteria, was recently revised into the Rome IV criteria. The Rome IV criteria define IBS as abdominal pain with altered bowel habits as the predominant symptom at least once a week in the last 3 months [8]. The term abdominal pain replaces the prior term of “discomfort”, which was deemed to be nonspecific and lacking in cultural sensitivity as it has variable meanings in different languages. Additionally, the Bristol Stool Scale is used for subtyping IBS according to stool form and frequency into its predominant bowel habit [8]. Despite extensive research, the pathophysiology of IBS remains elusive. Various mechanisms have been proposed including increased visceral sensitivity, alterations in intestinal motility and permeability, immune dysfunction, autonomic nervous system dysregulation, alterations in the gut microbiome, brain-gut axis alterations, and genetic and psychosocial factors [2].

The role of genetics in IBS has been investigated in pedigree, genome-wide association, and international twin studies [9–19]. In addition to identifying genetic risk loci for IBS, genetic polymorphisms have been associated with specific phenotypic characteristics of the syndrome [9, 10, 20–22]. Studies suggest that environmental triggers, including bacterial infection, air and water pollution, and radiation contribute to the development of IBS presumably in a susceptible person [23, 24]. Research suggests that environmental pollutants alter microbiome composition, an increasingly recognized plausible factor in IBS etiology [23, 25, 26]. Because the GI tract is sensitive to particulate matter (PM) and smoking, exposure to air pollution may lead to oxidative damage of colonic mucosa or exacerbate inflammation, presenting a risk factor for IBS [23, 24, 27, 28].

Beyond genetic and environmental factors, patients with more severe forms of IBS report higher psychosocial problems [13, 29–34]. Anxiety and depression occur in a significantly greater number of patients with IBS compared to patients without IBS [35, 36]. A positive correlation has been shown between anxiety, depression, and the severity of IBS symptoms [13, 37]. Recent work has shown that the application of cognitive behavioral therapy (CBT) can significantly reduce reported symptom severity, reduce pain severity, and improve the quality of life in patients with IBS [38].

Interestingly, the association between IBS and psychosocial problems may be accounted for due to alteration in the intestinal microbiota in IBS patients. Certain taxa of microbiota have been associated with specific IBS symptoms, while IBS symptom severity correlates with gut microbiota instability [13, 39–42]. For example, the bacterial family Actinomycetaceae was less prevalent in the GI tracts of IBS patients that are depressed compared to IBS patients who do not have depression [13]. Another example is how the distribution of gut bacterial phyla Clostridiales has been observed to distinguish IBS subtypes [43].

Moreover, evidence suggests impaired serotonergic signaling in IBS patients [44]. Serotonin (5-hydroxytryptamine, 5-HT) is one of the most-studied neurotransmitters in IBS due to its abundance within cells throughout the GI tract (~95%) and its role in the brain-gut axis [44–46]. Enterochromaffin cells (EC cells) synthesize approximately 90% of serotonin, with the remainder produced within the myenteric plexus [47]. Patients with IBS have more EC cells than controls [44, 48, 49]. Serotonin is secreted by EC cells via a variety of stimuli and plays a pivotal role in modulating the immune system and pathophysiological responses such as intestinal muscle contractility and inflammatory responses [13, 50]. Serotonin transporter (SERT) and tryptophan hydroxylase-1 (TPH1), the rate-limiting enzyme in serotonin production, is found in low levels in patients with IBS [51]. Other studies have shown disrupted enteric serotonergic signaling in IBS [52].

A specific connection between serotonergic signaling and IBS is not clear. However, the use of selective serotonin reuptake inhibitors (SSRIs) as a SERT antagonist employed in the treatment of depression, has also been shown to alleviate IBS symptoms [53]. The biosynthetic pathway of serotonin is a multistep process with tryptophan hydroxylase (TPH) acting as the rate-limiting enzyme. Evidence suggests that the expression of TPH1, the main isozyme of tryptophan hydroxylase, might be modulated by dihydroxycholecalciferol (vitamin D) [54]. In another study, tryptophan hydroxylase was stimulated by calcitriol (1,25D), the active hormonal vitamin D metabolite, in serotonergic neurons [55]. Vitamin D plays a crucial role in inflammatory processes and immunomodulatory activity [56]. Moreover, vitamin D deficiency has been associated with depression, a significantly reported co-morbidity among IBS patients, particularly those with IBS-D [13, 57, 58]. The role of vitamin D in the pathogenesis of IBS is emerging, and vitamin D supplementation has been proposed as a potential treatment for IBS [13, 59–62].

Several studies have attempted to identify specific candidate genes involved in the pathogenesis of IBS [44]. Some studies have shown dysregulated serotonergic signaling and blood serotonin levels [44]. Patients with the IBS-D subtype have higher blood serotonin levels [44, 63], while lower blood serotonin levels occur in the IBS-C subtypes [44, 52, 64]. More specifically, an analysis in blood and mucosal biopsies of 5-HT/5 HIAA ratios suggested defects in serotonin release in IBS-C subtypes and irregularities in serotonin uptake in IBS-D [44, 52, 64]. The *SERT* gene, also known as *SLC6A4*, has been studied extensively in relation to IBS; however, there is a lack of consensus regarding the association between IBS and *SERT* [44, 51, 65, 66].

In related GI disorders such as IBD, DNA microarrays of colonic tissue have successfully identified variants associated with the disease states [67]. Similarly, DNA microarrays have been used with IBS samples to identify distinguishing genetic characteristics between IBS and non-IBS individuals, but with less successful results. In this present study, several genes from our previous work using DNA microarrays and gene ontology (GO) analysis were screened using RT-qPCR to identify distinguishing biomarkers and a unique gene expression profile that may elucidate the pathological state of IBS [68]. Such a gene expression profile might serve as a foundation for the development of a diagnostic panel for IBS. Additionally, we sought to ascertain the effect of active vitamin D (1,25D) in HCT-116 human colorectal cells in regulating key genes associated with IBS. We propose that the current study may help improve the efficiency of IBS diagnosis, as well as identify novel genes involved in IBS pathogenesis, thus leading to a more timely and efficient method of disease management along with more effective targeted treatments.

## Methods

### Patient cohort characteristics

Patients were recruited for participation from a rural Midwestern multispecialty group practice. Mucosal tissue samples were collected from the sigmoid colon of 29 participants in the manner described by Costello et al. [67]. The experimental group consisted of 11 patients (eight females and three males) ranging from 41 to 58-years-old diagnosed with IBS-C, IBS-D, or IBS-M using the Rome III criteria. The control group included 18 patients (12 females and six males) ranging from 47 to 60-years-old, who did not meet diagnostic Rome III criteria for IBS. Patients enrolled in this study provided written informed consent. All experimental procedures were approved by the Marshfield Clinic Research Foundation and Arizona State University Institutional Research Board (#YAL10308 and #1106006538 respectively) in accordance with institutional and national research committee ethical standards.

### Gene expression profiling and ontology enrichment analysis

Mucosal tissue samples from the sigmoid colon of IBS and control patients were processed for DNA microarray analysis and GO pathway enrichment as previously described [68]. Differential expression of 858 genetic features between the two experimental groups and GO terms with a p-value <0.05 were identified and scrutinized for further investigation in this study.

### Tissue: RNA extraction, purification, and quality control

Colonic tissue samples were weighed and then suspended in 700 μL of lysis buffer. An ultrasonic converter was placed on ice for three minutes, rinsed with DI water, and dried with a Kimwipe followed by 5 x 10 seconds of sonication on ice (setting 3 on Fisher Scientific Sonic Dismembrator, Model 100). The ultrasonic converter was placed on ice for 30 seconds between sonication rounds to avoid specimen degradation. RNA was extracted and purified under RNase-free conditions in accordance with the manufacturer’s protocol for the Aurum Total RNA Mini kit (Bio-Rad, Hercules, CA). A spectrophotometer (Eppendorf BioPhotometer) was employed to obtain A260/280, A260/230 ratios and concentrations for analysis of RNA quality and yield.

### Cell culture: RNA extraction, purification, and quality analysis

Human colorectal cancer cells (HCT-116) were obtained from the American Type Culture Collection in Manassas, VA, and used to investigate fold-induction by 1,25-dihydroxyvitamin D_3_ (1,25D) on IBS biomarkers of interest in colonic epithelial cells. The complete growth medium used contained DMEM/high glucose (including 4.00 mM L-glutamine, 4500 mg/L glucose, and sodium pyruvate) supplemented with 10% fetal bovine serum and 1X penicillin/streptomycin. Cells were plated at 800,000 per well, induced with either 10nM (1,25D) or ethanol (vehicle control), and incubated at 37°C with 5% CO_2_ air atmosphere for 24 hours. Extraction and purification of RNA were conducted under RNase-free conditions and in accordance with the manufacturer’s protocol of the Aurum Total RNA Mini kit (Bio-Rad, Hercules, CA). A spectrophotometer (Eppendorf BioPhotometer) was employed to assess RNA quality and yield.

### RT-qPCR of sample tissue and HCT-116 cells

RT-qPCR was employed to examine the gene expression of potential IBS biomarkers. The results were compared to the control cohort to test the external validity of the DNA microarray data. RNA was extracted and purified from patient mucosal tissue samples and HCT-116 cells. Spectroscopy was employed to obtain A260 and A280 values to assess RNA yield and quality. Next, 1 μg RNA was reverse transcribed to produce 20 μL of first-strand complementary DNA (cDNA) using the iScript cDNA Synthesis Kit (Bio-Rad). Reaction mixtures for RT-qPCR comprised 1.5 μL of cDNA, 3.5 μL of the appropriate primer, and 5 μL of SYBR Green Master Mix (Roche Applied Science, Indianapolis, IN, USA). Various aggregate mixtures of pooled control samples were tested for experimental optimization and reproducibility. The control RNA used in this study was an RNA aggregate of eight patients who did not meet the diagnostic criteria for IBS. The RT-qPCR data for each gene of interest is reported as a global standard error mean (SEM) relative to pooled non-IBS patients. Gene percent accuracy is reported for suggested IBS biomarkers for a diagnostic assay using Eq 1. For HCT-116 cells, the RT-qPCR data are reported as a fold change of gene expression in the presence of the active form of vitamin D (1,25D) compared to the expression in cells dosed with an ethanol vehicle control.


x=No.ofpatientsthatmatchthegeneexpressionoftheIBSmicroarrayinagiventechnicalreplicate

y=TotalnumberofIBSpatientsanalyzedinagiventechnicalreplicate

$$Gene\;\%\;Accuracy=\frac{\sum \frac{x}{y}}{No.\;of\;Replicates}*100$$ (1)

### Statistical analyses

All genes reported in Table 2 had a minimum of two independent RT-qPCR experiments (and usually 3 or more) performed with a minimum of three to six replicate PCR wells per patient and gene when analyzing IBS gene expression, with "fold change" computed as experimental (IBS) group relative to control (non-IBS) patients. All analyses were performed in a blinded fashion. Agilent’s Feature Extraction software (v. 11.0.1.1) was utilized to process DNA microarray data and normalized using limma (Ritchie et al. 2015) [69]. The data were used to identify differentially expressed genes through a combination of limma linear model t-test, Mann-Whitney U-test, and two-tailed student’s t-test with a *p*-value ≤ 0.05 considered statistically significant.

## Results

### Gene expression profiling and serotonergic pathways in IBS patients

**Fig 1:**
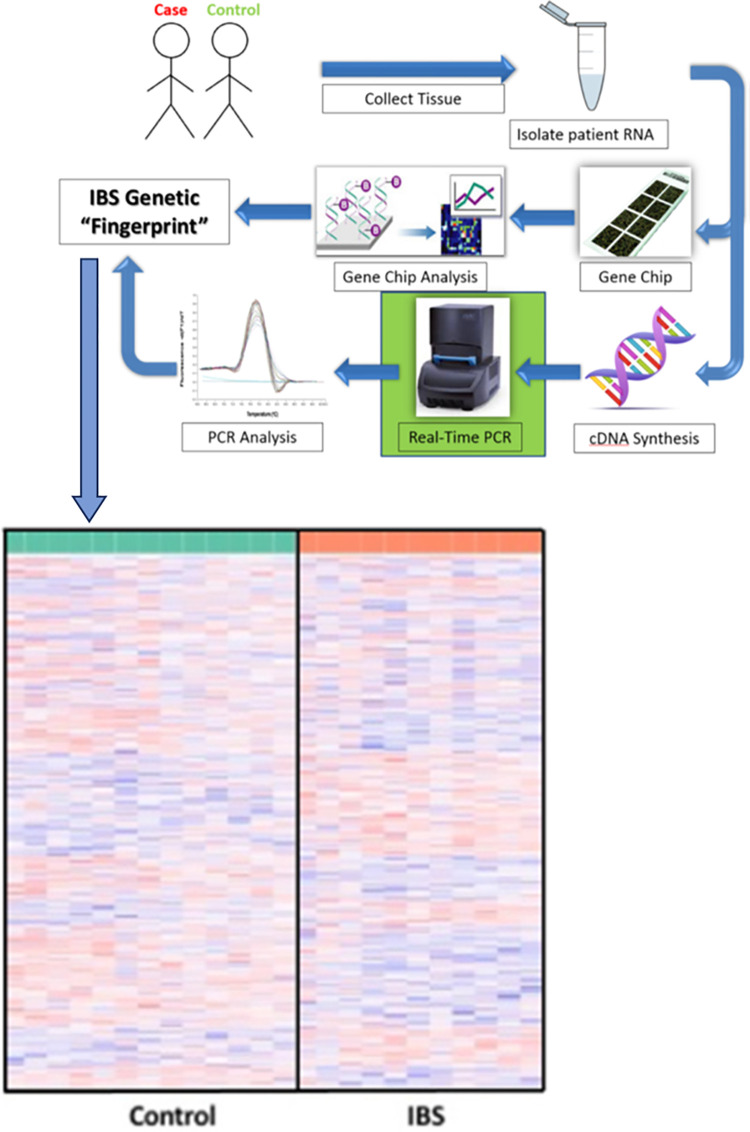
Workflow and DNA microarray analysis. Workflow in the acquisition of an IBS genetic “fingerprint” from patient sample to transcriptomic analysis. Differentially expressed genes from mucosal tissue biopsy samples in the sigmoid colon of patients diagnosed with irritable bowel syndrome (IBS) and non-IBS patients (control). The heat map (adapted from Dussik et al.) displayed differential expression of 858 genetic features between the two experimental groups (p-value < 0.05) [68]. Pronounced clusters of similar color and intensity are also visible with blue indicative of individual biomarkers with lower expression levels and red illustrating higher levels of expression.

Mucosal tissue biopsy samples in the sigmoid colon of patients diagnosed with IBS and non-IBS patients (control) were profiled using DNA microarray analysis. Patient demographics are outlined in Table 1. As previously shown, 858 genetic features were identified with differential expression levels between IBS patients and patients not diagnosed with IBS [68]. Statistical analysis further narrowed the results of the DNA microarray results to 200 differentially expressed genes between the groups to be externally validated by RT-qPCR (Fig 1). GO analysis of these identified genetic features revealed that among the top 25 (ranked by increasing p-value) GO terms, seven were involved with serotonin metabolism [68].

**Table 1:** Demographical and IBS subtype designations of study population.

Sample ID	Classification	IBS Subtype	Sex	Age
IBS-006	Control	-	F	55
IBS-011	Control	-	F	50
IBS-012	Control	-	F	50
IBS-016	Control	-	F	52
IBS-017	Control	-	F	57
IBS-021	Control	-	F	49
IBS-022	Control	-	F	60
IBS-032	Control	-	F	50
IBS-043	Control	-	F	54
IBS-045	Control	-	F	50
IBS-046	Control	-	F	51
IBS-056	Control	-	F	51
IBS-010	Control	-	M	54
IBS-019	Control	-	M	50
IBS-020	Control	-	M	47
IBS-026	Control	-	M	53
IBS-027	Control	-	M	50
IBS-030	Control	-	M	47
IBS-002	Case	IBS-D	F	56
IBS-003	Case	Unknown	F	49
IBS-004	Case	IBS-M	F	51
IBS-007	Case	IBS-M	F	53
IBS-009	Case	IBS-D	M	58
IBS-013	Case	IBS-C	F	57
IBS-024	Case	IBS-M	M	51
IBS-025	Case	IBS-D	M	55
IBS-028	Case	IBS-D	F	55
IBS-033	Case	IBS-M	F	46
IBS-034	Case	IBS-M	F	41

IBS-D, irritable bowel syndrome subtype diarrhea; IBS-C, irritable bowel syndrome subtype constipation; IBS-M, irritable bowel syndrome mixed subtypes.

### Potential IBS biomarkers

**Fig 2:**
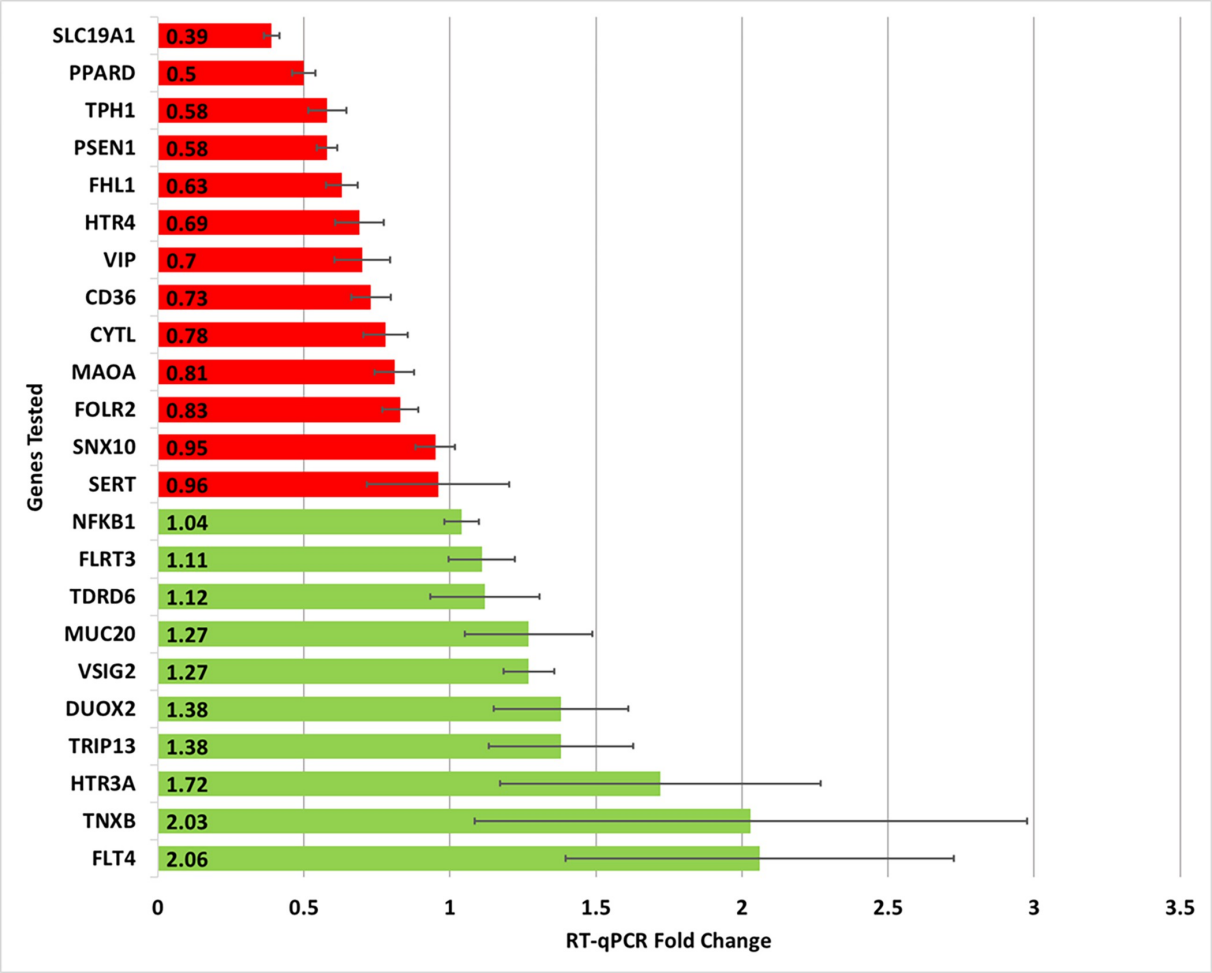
Gene expression fold change in IBS patients relative to control patients using RT-qPCR testing. In total, 29 genes of interest were evaluated with only 23 being reported here (only those genes with at least two independent RT-qPCR plate replicates are included). Red coloration is indicative of lower levels of expression while green represents higher expression levels with SEM being reported.

The genes selected for further analysis by RT-qPCR were identified based on the following criteria: significance in the GO analysis, microarray statistical significance, function in relation to IBS pathophysiology, and high differential expression between the two groups in gene expression profiling. As a result of these criteria, 29 genes were selected and tested using RT-qPCR in 11 IBS-derived RNA, as well as control RNA from an aggregate of eight asymptomatic patients (S1 Table). From the initial 29 selected genes, the 23 reported in Fig 2 have a minimum of two independent RT-qPCR plate replicates. Next, RT-qPCR-measured fold change of the filtered 23 genes was compared to DNA microarray fold change followed by calculation of percent accuracy for each replicate per gene calculated using Eq 1 (Table 2). Of the 23 filtered genes tested using RT-qPCR, only genes with an overall minimum 70 percent accuracy and function related to IBS pathophysiology were considered for a proposed IBS biomarker assay. As a result of these criteria, seven genes of interest were identified as potential IBS biomarkers with an average accuracy of 84.2% (Table 2).

**Table 2:** Putative IBS biomarker genes with calculated accuracy using DNA microarray and RT-qPCR as a validation tool.

Gene	Function	IBS Microarray Fold Change	RT-qPCR Fold Change (Fig 2)	% Accuracy
***SLC19A1***	Involved in vitamin B9 metabolism. High level of expression in placenta and the testis. Lower levels of expression in small intestine and colon	0.62	0.39	100
***PPARD***	Target of non-steroidal inflammatory drugs, expressed in preadipocytes and in low levels in the colorectal mucosa	0.70	0.50	92.42
***TPH1***	Encodes the enzyme involved in the rate-limiting step of serotonin synthesis. Expressed in smooth muscles in the small intestines and colon	0.93	0.58	84.85
***CD36***	Protein- coding receptor for thrombospondin in platelets. Expressed in different types of epithelial and endothelial cells. Co-receptor that stimulates inflammatory response and the NFKB pathway	0.64	0.73	81.82
***VIP***	Precursor of the vasoactive intestinal polypeptide. Causes vasodilation and relaxes the smooth muscle of the stomach and gallbladder	0.74	0.70	81.82
***SERT***	Serotonin transporter involved in the central nervous system via reuptake of serotonin from the synaptic cleft to the pre-synaptic terminal for reuse	0.48	0.96	75.76
***VSIG2***	Involved in the metabolic pathway and synthesis of amino acids. Low levels of expression found in the intestinal tract	1.14	1.27	72.73

*SLC19A1*, Solute Carrier Family 19 Member 1; *PPARD*, Peroxisome Proliferator Activated Receptor Delta; *TPH1*, Tryptophan Hydroxylase 1; *CD36*, CD36 Molecule; *VIP*, Vasoactive Intestinal Peptide; *SERT*, Sodium-Dependent Serotonin Transporter; *VSIG2*, V-Set and Immunoglobulin Domain Containing 2.

### Vitamin D-meditated regulation of IBS biomarkers of interest in human colorectal cells

**Fig 3:**
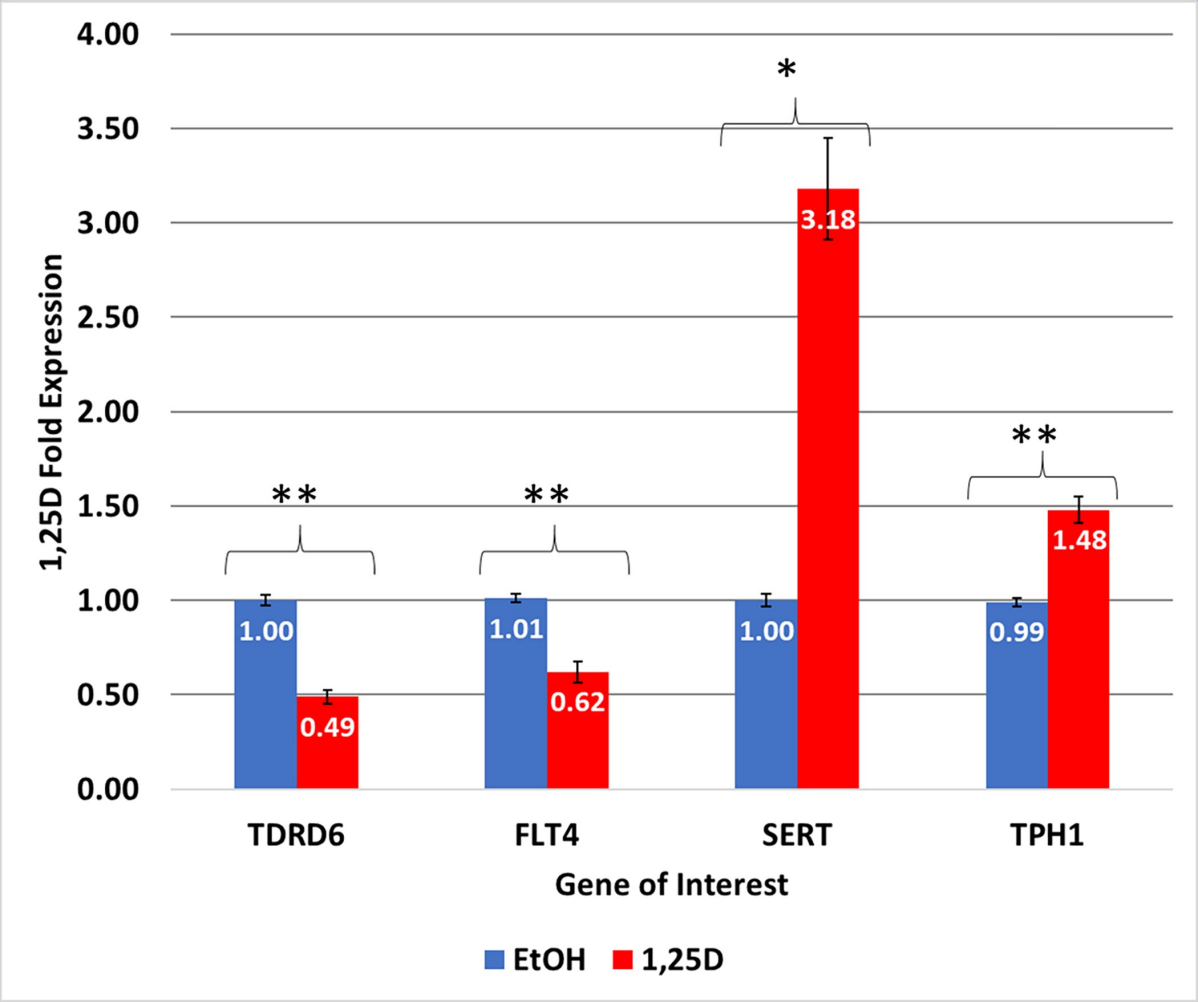
IBS-Associated gene regulation by 1,25D. The mRNA levels of each gene were determined using quantitative real-time polymerase chain reaction (RT-qPCR) for a total of three to six replicates. Values are reported as fold change relative to pooled control. SEM reported on bar plots. Ranges of p-values are represented as: *P < 0.005; **P < 0.0005, t-Test: Two-Sample Assuming Equal Variance.

Given the prevalence of significantly altered serotonergic-related pathway expression from GO analysis, serotonin’s close association with the GI tract, and the positive correlation between vitamin D and serotonin synthesis via *TPH1* induction, a selection of differentially expressed genes in IBS patients (Fig 2) was assessed for regulation by vitamin D in human colonic cells [45, 70, 71]. HCT-116 colorectal cells were treated with 10^−8^ M 1,25D for 24 hours, and RT-qPCR was employed to analyze mRNA expression levels of four potential IBS genetic biomarkers (Fig 3). The four specific IBS-candidate genes were selected using microarray data based on significance (p < 0.05) and differential regulation in IBS patients relative to controls as indicated by the RT-qPCR data (Fig 2). *TPH1* and *SERT* were selected based upon their association with serotonin (and thus vitamin D), while *TDRD6* and *FLT4* were selected to provide a complete spectrum of differential gene expression and to further validate and extend our previous data of these potential IBS biomarkers [68]. As shown, *TPH1* and *FLT4* represent more extreme repression and induction, respectively, while *SERT* and *TDRD6* displayed more moderate levels of repression and induction relative to the controls (Fig 2). All IBS biomarkers tested for regulation by vitamin D appeared influenced by the presence of 10^−8^ M 1,25D. More specifically, gene regulation by vitamin D resulted in *TDRD6* repression by 0.49-fold (*p* < 0.0005), *FLT4* repression by 0.62-fold (*p* < 0.0005), *SERT* induction by 3.18 (p < 0.005), and *TPH1* induction by 1.48-fold (*p* < 0.0005) (Fig 3). RT-qPCR patient data indicated an induced expression pattern in *TDRD6* and *FLT4*, while TPH1 and *SERT* exhibited a repressed expression pattern in the IBS cohort (Fig 2).

## Discussion

DNA microarray analysis revealed 858 genetic features with differential expression between IBS and control patient populations, providing evidence that IBS pathophysiology may be linked to and/or reflected in the transcriptome [72, 73]. Differential expression of genes in IBS patients better clarifies the IBS pathophysiological state. Therefore, GO interaction analysis was performed to assess patterns of differentially expressed genes in IBS on biochemical pathways to identify which pathways might be disrupted or modified in the IBS disease state. Differentially expressed genes between the IBS and the control cohort were grouped into pathways that consisted of five or more genes with similar functions and/or shared interactions. The pathways most influenced by IBS were serotonin uptake, positive regulation of glycogen (starch) synthase activity, negative regulation of serotonin secretion, SA node cell action potential, and Type II Na+/Pi co-transporters. The serotonergic pathway was most prevalent among differentially expressed genes, consistent with the role that serotonin metabolism may play a role in the pathogenesis of IBS [68]. This association between IBS and serotonin is supported as some serotonergic agents have been successfully implemented in evidenced-based therapeutic regimes in IBS [74, 75].

In total, 29 genes were identified for more extensive testing based upon the following criteria: differential expression between IBS patients and control in the DNA microarray, GO analysis, and primary gene function (with an emphasis on serotonergic signaling/metabolism). To validate data from DNA microarray, RT-qPCR was employed. Differentially expressed genes between IBS and control patients identified by DNA microarray analysis were compared to the RT-qPCR measured fold change in colonic tissue from 11 IBS patients compared to non-IBS patient controls to calculate a percent accuracy for each gene. RT-qPCR analyses revealed seven genes with expression patterns sufficiently consistent to indicate the viability of an IBS diagnostic tool. The seven potential genes, six repressed and one up-regulated (underlined), were *SLC19A1*, *PPARD*, *TPH1*, *CD36*, *VIP*, *SERT*, and *VSIG2*. Collectively, these putative genetic biomarkers could be used to more efficiently screen for IBS with an average accuracy of 84.2% in our blinded analysis (Table 2).

It is interesting to note that the IBS microarray data suggests repression of *MUC20* and induction of *FOLR2*, *MAOA*, *HTR4*, *FHL1*, *PSEN1*, *MMP26*, and *GC* while further investigation with RT-qPCR contradicted the preliminary microarray data (S1 Table). The use of RT-qPCR as a validation tool for microarray analysis suggests that *MUC20* is induced and *FOLR2*, *MAOA*, *HTR4*, *FHL1*, *PSEN1*, *MMP26*, and *GC* are in fact repressed in IBS patients. However, an important note is that the fold changes of *MMP26* and *GC* as described by RT-qPCR were calculated from a single plate replicate (S1 Table) and warrant further study before a clear expression pattern in IBS patients is identified.

Associations between IBS subtypes and *SERT* polymorphisms have also been studied [44, 76, 77]. A meta-analysis that included 12 studies and 2,068 IBS patients did not find an association between *SERT* polymorphisms and IBS [78]. However, another meta-analysis of 25 studies and 3,443 IBS patients did find a positive association between IBS and *SERT* polymorphisms, but only among East Asian and not Caucasian populations [79]. TPH, the rate-limiting enzyme that catalyzes serotonin biosynthesis, has two isoforms: TPH1 and TPH2. There appears to be an association between TPH1 and IBS severity [44]. Additionally, colonic mucosa levels in IBS patients have been found to have significantly reduced TPH1 mRNA levels [44]. Given the apparent importance of *TPH1* and *SERT* in relation to the IBS disease state, both genes were a primary focus for testing.

The expression level of *TPH1*, one of the main isoforms of tryptophan hydroxylase, was among the genes explored using RT-qPCR to determine whether IBS patients exhibited repressed GI serotonin production by indirect means. In the IBS cohort, 10 out of 11 patients showed decreased levels of *TPH1* when compared to controls [68]. There is evidence to suggest vitamin D plays a role in controlling the rate of serotonin synthesis *in vivo* by modulating the expression of *TPH1* [54]. Indeed our group found that approximately 82 percent of sampled patients with IBS exhibited serum 25-hydroxyvitamin D levels below the optimal clinical level [68]. Expanding upon this, we hypothesized that the active hormonal metabolite of vitamin D, 1,25-dihydroxyvitamin D3 (1,25D), may influence the expression of other putative IBS candidate genetic biomarkers, similar to the role of vitamin D in modulating TPH1 expression.

To test the effect of vitamin D, HCT-116 colorectal cells were treated with 10^−8^ M 1,25D for 24 hours, and RT-qPCR was employed to test the expression patterns of four candidate IBS genes (*TDRD6*, *FLT4*, *SERT*, and *TPH1*). As previously described, *TPH1* and *SERT* were selected for their role in serotonergic metabolism, and *TDRD6* and *FLT4* were chosen to provide a spectrum of varying expression levels in IBS patients. Specifically, *TPH1* and *FLT4* represented greater differential expression levels in repression and induction, respectively in IBS patients relative to control. Meanwhile, *SERT* and *TDRD6* represented more moderate differential expression in repression and induction respectively (Fig 2). Vitamin D significantly regulated the expression of all four genes in the opposite direction than what was observed in the IBS cohort. Significantly, the reversal of gene expression in human colonic cells by 1,25D relative to IBS patients in both the microarray and RT-qPCR data suggests that vitamin D may be effective at reversing biomarker gene expression in IBS patients and thus alleviate symptoms (Figs 2 and 3).

Serotonin secretion by enterochromaffin cells is correlated with GI motility via smooth muscle contractility [46, 80–82]. In the context of IBS, IBS-D patients would be associated with elevated serotonin production, IBS-C with depressed serotonin production, and IBS-M with alternating or intermediate serotonin production. The IBS cohort of this study consisted of one IBS-C patient, one unknown, four IBS-D patients, and the remaining IBS-M. Due to the cohort consisting of only one IBS-C patient, and four IBS-D patients, we expected to see intermediate to elevated serotonin production as an average. However, approximately 90 percent of the IBS patient tissue exhibited repressed *TPH1* expression levels relative to controls.

The significance of this finding can be explained based on the analysis conducted by Sabir et al. They measured serotonin production in response to vitamin D in RN46A-B14, a serotonergic cell line [70]. The authors reported induction of *TPH1* mRNA and the increased synthesis of serotonin by vitamin D, suggesting that 5-HT production occurs in response to vitamin D in the large intestine. Reduced levels of *TPH1* in IBS-D patients result from negative feedback by enteric 5-HT signaling, while decreased *TPH1* levels in other IBS subtypes may be due to vitamin D insufficiency. Further support is based on a finding in our study of increased levels of expression of *SERT* and *TPH1* in HCT-116 colorectal cells when treated with 1,25D. We previously reported a trend of decreased serum vitamin D levels in IBS patients relative to the control cohort [68]. Therefore, the irregular serotonin production that generally describes the IBS pathological state may be a result of vitamin D deficiency.

Tazzyman et al. reported decreased serum 25(OH)D levels among IBS patients and noted a positive correlation between vitamin D supplementation and quality of life [83]. The authors found that IBS-C patients were the most responsive to the vitamin D supplementation resulting in improvement across nearly all IBS symptoms. Since IBS-C patients are thought to have diminished serotonin production, the responsiveness of IBS-C patients to vitamin D is consistent with vitamin D increasing *TPH1* serotonin levels in the gut and our hypothesis that induction of the *TPH1* gene in the gut by vitamin D may play a role in IBS pathophysiology [55, 84].

Though Tazzyman et al. reported a positive correlation between vitamin D supplementation and quality of life, the contradictory findings reported by Williams et al. found no improvement in IBS symptoms with vitamin D supplementation [83, 85]. Differences in measuring vitamin D in serum blood samples via immunoassay and dry blood spotting via LC-MS-MS assay could explain these contradictory findings. Our study suggests that, in a controlled, in vitro model, the active metabolite of vitamin D is capable of reversing potentially aberrant/pathological IBS gene expression with emphasis on serotonergic metabolism given the GO analysis highlighting serotonergic signaling and the markedly significant increase in *SERT* and *TPH1* expression in HCT-116 colorectal cells when treated with 1,25D. It is important to note that all four genes tested for regulation by vitamin D were influenced by the presence of 10^−8^ M vitamin D. Thus, these findings may inform future treatment decisions and research into patients diagnosed with IBS.

Of particular note in this study was the analysis of gene expression by RT-qPCR in IBS patients relative to controls, evaluation of the accuracy of the proposed biomarkers, and assessment of the regulatory role of vitamin D in genes associated with serotonin metabolism. Outside of reversal of aberrant gene expression by vitamin D, we have also identified seven putative biomarker genes that may be of use in the diagnosis of IBS. Building the bridge between clinical and nonclinical uses of vitamin D in the treatment of IBS and serotonergic signaling will likely require further research not only at the genomic and transcriptomic levels, but also in the proteomic, metabolomic, and microbiome realms of IBS pathophysiology via a multiomic approach [68, 86]. Limitations of this study include a small IBS patient sample of varying subtypes rather than of a single subtype. Such nonspecific sampling may lead to trends in data that are not reflected among a single IBS subtype, thus obscuring the development of more targeted therapies. Future work should focus on such multiomics-based approaches to understanding IBS and an expansion of sample pools to include greater patient numbers and diversity to enable subsampling and analysis of multiple IBS subtypes.

## Supporting information

S1 Table Complete 29 genes tested by RT-qPCR. Complete 29 genes tested in RT-qPCR analysis compared to IBS Microarray values. Genes with a minimum of 2 replicates are graphically reported in Fig 2. Fold change reported in IBS Microarray and RT-qPCR data relative to pooled control patients.
(DOCX)
