# Supplementary material for: Identification of putative transcriptomic biomarkers in irritable bowel syndrome (IBS): Differential gene expression and regulation of TPH1 and SERT by vitamin D
Source: PLoS One. 2022 Oct 20;17(10):e0275683. doi: 10.1371/journal.pone.0275683 (PMC9584396; doi:10.1371/journal.pone.0275683)
Supplement: S1 Table — Complete 29 genes tested in RT-qPCR analysis compared to IBS Microarray values. Genes with a minimum of 2 replicates are graphically reported in Fig 2. Fold change reported in IBS Microarray and RT-qPCR data relative to pooled control patients. (DOCX) [file pone.0275683.s001.docx]

**S1 Table. Complete 29 genes tested by RT-qPCR.**

| **Gene** | **IBS Microarray Fold Change** | **RT-qPCR Average Fold Change** | **No. of RT-qPCR Replicates** | **RT-qPCR ± Standard Error** |
| --- | --- | --- | --- | --- |
| SLC9A4 | 1.56 | N/A | 0 | N/A |
| SLC6A11 | 11.05 | N/A | 0 | N/A |
| GABRR1 | 8.81 | N/A | 0 | N/A |
| HAS1 | 7.45 | 2.64 | 1 | 0.19 |
| FLT4 | 1.18 | 2.06 | 2 | 0.66 |
| TNXB | 1.48 | 2.03 | 4 | 0.95 |
| HTR3A | 1.45 | 1.72 | 2 | 0.55 |
| TNFSF18 | 6.7 | 1.44 | 1 | 0.3 |
| TRIP13 | 1.27 | 1.38 | 3 | 0.25 |
| DUOX2 | 1.68 | 1.38 | 2 | 0.23 |
| VSIG2 | 1.14 | 1.27 | 2 | 0.09 |
| MUC20 | 0.94 | 1.27 | 2 | 0.22 |
| TDRD6 | 16.07 | 1.12 | 3 | 0.19 |
| FLRT3 | 1.54 | 1.11 | 3 | 0.11 |
| NFKB1 | 1.4 | 1.04 | 5 | 0.06 |
| MBD2 | 0.47 | 0.96 | 1 | 0.09 |
| SERT | 0.48 | 0.96 | 3 | 0.24 |
| SNX10 | 0.33 | 0.95 | 3 | 0.07 |
| FOLR2 | 1.28 | 0.83 | 4 | 0.06 |
| MAOA | 1.04 | 0.81 | 3 | 0.07 |
| CYTL | 0.68 | 0.78 | 4 | 0.08 |
| CD36 | 0.64 | 0.73 | 4 | 0.07 |
| VIP | 0.74 | 0.7 | 4 | 0.09 |
| HTR4 | 1.05 | 0.69 | 2 | 0.08 |
| FHL1 | 1.48 | 0.63 | 3 | 0.05 |
| PSEN1 | 1.26 | 0.58 | 6 | 0.03 |
| TPH1 | 0.93 | 0.58 | 3 | 0.06 |
| MMP26 | 27.73 | 0.51 | 1 | 0.13 |
| PPARD | 0.7 | 0.5 | 6 | 0.04 |
| EPHA3 | 0 | 0.45 | 1 | 0.07 |
| GC | 8.38 | 0.4 | 1 | 0.2 |
| SLC19A1 | 0.62 | 0.39 | 6 | 0.03 |

Complete 29 genes tested in RT-qPCR analysis compared to IBS Microarray values. Genes with a minimum of 2 replicates are reported in Figure 2 (a total of 23 of the 29 genes are shown). Fold change reported in IBS Microarray and RT-qPCR data relative to pooled control patients.
